# SHSST Cyclodextrin Complex Prevents the Fibrosis Effect on CCl_4_-Induced Cirrhotic Cardiomyopathy in Rats through TGF-β Pathway Inhibition Effects

**DOI:** 10.3390/ijms15058037

**Published:** 2014-05-08

**Authors:** Cheng-Hsun Yang, Wei-Jen Ting, Cecilia Hsuan Day, Da-Tong Ju, Yu-Lan Yeh, Li-Chin Chung, Fu-Jenn Tsai, Chang-Hai Tsai, Yuhsin Tsai, Chih-Yang Huang

**Affiliations:** 1 Graduate Institute of Chinese Medicine, China Medical University, 91 Hsueh-Shih Road, Taichung 40402, Taiwan; E-Mail: littleraxg@gmail.com; 2 Graduate Institute of Basic Medical Science, China Medical University, 91 Hsueh-Shih Road, Taichung 40402, Taiwan; E-Mail: Wei-Jen.Ting@outlook.com; 3 Department of Nursing, Mei Ho University, 23 Pingguang Road, Pingtung 91202, Taiwan; E-Mail: x00003023@mail.meiho.edu.tw; 4 Department of Neurological Surgery, Tri-Service General Hospital, National Defense Medical Center, 325, Section 2, Chenggong Road, Neihu District, Taipei 114, Taiwan; E-Mail: wxyz670628@yahoo.com.tw; 5 Department of Pathology, Changhua Christian Hospital, 135 Nanxiao Street, Changhua 50006, Taiwan; E-Mail: 1867@cch.org.tw; 6 Department of Medical Technology, Jen-Teh Junior College of Medicine, 79-9 Sha-Luen Hu, Xi-Zhou Li, Hou-Loung Town, Miaoli 35664, Taiwan; 7 Department of Hospital and Health Care Administration, China Nan University of Pharmacy & Science, 60, Section 1, Erren Road, Rende District, Tainan 71710, Taiwan; E-Mail: alanjack@mail.chna.edu.tw; 8 School of Chinese Medicine, China Medical University, 91 Hsueh-Shih Road, Taichung 40402, Taiwan; E-Mail: fujenntsai@yahoo.com; 9 Department of Healthcare Administration, Asia University, 500 Lioufeng Road, Taichung 41354, Taiwan; E-Mail: changhaitsai@yahoo.com.tw; 10 Department of Health and Nutrition Biotechnology, Asia University, 500 Lioufeng Road, Taichung 41354, Taiwan

**Keywords:** San Huang Shel Shin Tang, silymarin, baicalein, cirrhotic cardiomyopathy

## Abstract

Patients with liver cirrhosis also have subtle cardiac structure or function abnormalities. This cardiac dysfunction commonly occurs in 56% of waiting orthotopic liver transplantation (OLT) patients and is defined as cirrhotic cardiomyopathy (CCM). Up to now, there is no standard treatment because CCM does not have a solidly established diagnosis and is based on high clinical suspicion. The liver function of CCM is particularly limited, making patients vulnerable to more drug treatments. Here, we use silymarin (100 mg/kg/day), baicalein (30 mg/kg/day), San Huang Shel Shin Tang (SHSST, 30 mg/kg/day) and β-cyclodextrin modified SHSST (SHSSTc, 30 and 300 mg/kg/day) treatments for a CCl_4_-induced CCM rat model. The results show that silymarin, baicalein and SHSST treatments can only slightly reduce the collagen accumulation in CCM rat hearts. However, SHSSTc treatment protects the heart in CCM and significantly inhibits collagen acumination and the fibrosis regulating transforming growth factor-β (TGF-β) pathway expression. SHSSTc treatments further reduced the heart weight and the ratio between left ventricular weight (LVW) and tibia length (TL). This experimental data show that water solubility improved β-cyclodextrin modified Chinese herbal medicine formula (SHSSTc) can provide an excellent heart protection effect through TGF-β pathway inhibition.

## 1. Introduction

Cirrhotic cardiomyopathy (CCM) was first described by Kowalski and Abelmann in 1953. The estimated incidence rate is about 56% of patients waiting for orthotopic liver transplantation (OLT) without a previous history of cardiac disease [1–4]. The CCM mechanism has been discussed relative to hyperdynamic circulation, but even after liver transplantation 7%–15% of deaths are related to cardiac-related dysfunction [5]. Humoral factor abnormalities are suggested to play important roles in cirrhosis, finally causing blunted cardiac response or cardiac dysfunction [6].

In liver fibrosis the transforming growth factor (TGF-β) is required and TGF-β signaling blunting can reduce fibrogenesis [7–9]. TGF-β production occurs in non-parenchymal liver cells when the liver is damaged, especially by Kupffer cells and hematopoietic stem cells (HSC) rather than fully differentiated epithelial cells [10]. In cardiac remodeling TGF-β leads in modulating fibroblast phenotype and gene expression [11]. TGF-β also promotes extracellular matrix deposition by upregulating collagen and fibronectin synthesis in the infarct [12]. In addition, dilative ventricular remodeling by inducing interstitial fibrosis is also mediated through the TGF-β signaling pathway [12].

Up to now, there has been no standard treatment because CCM does not have a solidly established diagnosis and is based on high clinical suspicion [13]. In the therapeutic strategy for fibrogenesis prevention, TGF-β signaling should also be reduced using any treatment for CCM patients [14–16]. Here, silymarin and a traditional Chinese herbal medicine formula (San Huang Shel Shin Tang, SHSST) and its water-soluble beta-cyclodextrin (β-CD) complex modification compound (SHSSTc) were used in treating CCM rats, which were induced from carbon tetrachloride (CCl_4_) intraperitoneal (IP) injection induced cirrhosis model [17–19]. Silymarin is a well known drug against cirrhosis and is a cocktail-like herbal liver-protective drug with four flavonolignan isomers, silybin, silychristin, silydianin and isosilybin [20,21]. SHSST is also a cocktail-like traditional herbal decoction used for liver and heart protection in China. SHSST is composed of 50% *Rheum officinale* Baill, 25% *Scutellaria baicalnsis* Geprgi and 25% *Coptis chinensis* Franch in weight [22,23]. Rheum was reported to have a liver protection effect that can protect the liver in CCl_4_-induced injury treatment in rats. *Scutellaria* and *Coptis* were also reported to have similar liver protection effects in acute hepatotoxicity. The liver protection effects between *Rheum*, *Scutellaria* and *Coptis chinensis* are due to the same bioactive compounds, baicalein and other flavonoids [24–28].

Both SHSST and silymarin are potential liver protection drugs, but both present poor water solubility and poor bioavailability [17]. A formulation approach is necessary to increase the solubility of these liver protection drugs. β-CD modification can increase the solubility and spectral properties of guest molecules, especially hydrophobic drugs, without changing their intrinsic property to permeate the cell membranes. Thus, SHSST was modified into SHSST-β-CD-complex (SHSSTc) and evaluated for its therapeutic effects in a CCM animal model.

## 2. Results and Discussion

The heart phenomena changes in each group were measured and presented in Table 1. The average heart weight in CCl_4_-induced CCM groups was higher than the control. The average heart weights in silymarin, baicalein, SHSST, and SHSSTc low and high dose treatments were reduced and similar to the control group average heart weight. The ratio between left ventricular weight (LVW) and tibia length (TL) is a particularly accurate indicator for cardiomegaly. The average LVW/TL of CCl_4_-induced CCM groups are higher, and silymarin and baicalein can slightly reduce cardiac hypertrophy. Interestingly, the SHSST, SHSSTc low and high dose treatments can significantly reverse cardiac hypertrophy in CCl_4_-induced CCM rat hearts.

**Figure 1.**
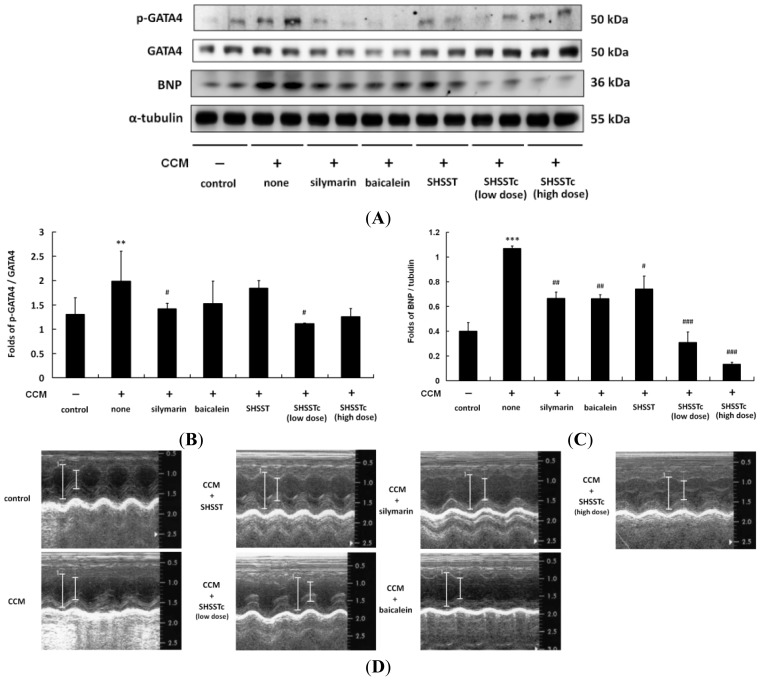
Phoshphorylated GATA binding protein 4 (p-GATA4) and brain natriuretic peptide (BNP) expressions in cirrhotic cardiomyopathy (CCM) hearts. (**A**) The p-GATA4 and BNP expressions (*n* = 6 in each group) were increased in CCM and reduced by silymarin, baicalein, SHSST (San Huang Shel Shin Tang), and SHSSTc low dose and high dose treatments; (**B**) The normalized protein expression folds of p-GATA4 with GATA4; (**C**) The normalized protein expression folds of BNP with α-tubulin (the scale bars is also presented as the normalized folds with α-tubulin); (**D**) Echocardiography analysis images (*n* = 6 in each group) of the heart function is compared by the left ventricular systolic and diastolic distance (cm). ** *p* < 0.01, *** *p* < 0.001 compared with control group; # *p* < 0.05, ## *p* < 0.01, ### *p* < 0.001 compared with CCM group.

In CCl_4_-induced CCM heart proteins analysis, the high level expression of brain natriuretic peptide (BNP) presents a stress load on the beating heart. (Figure 1) After 4 weeks of silymarin, baicalein and SHSST treatments, BNP levels were slightly reduced and lower than that in the CCM only group. Low and high dose SHSSTc treatments efficiently inhibited the BNP secretion in a dose dependent manner.

**Figure 2.**
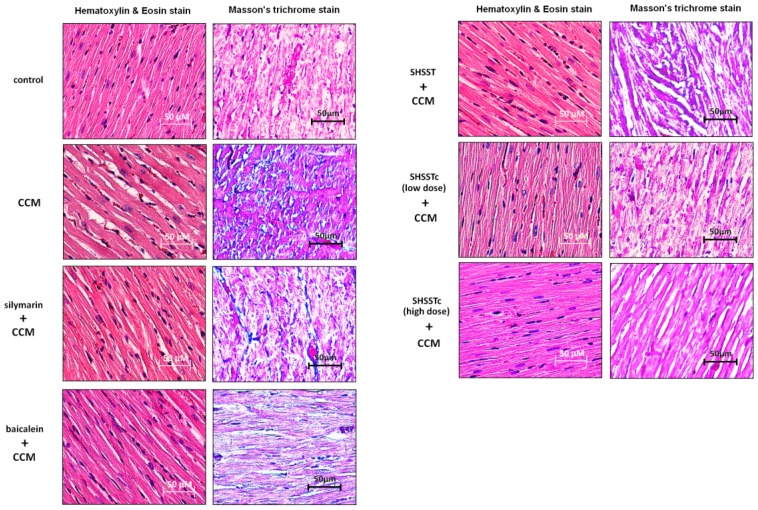
Morphology assessments by hematoxylin and eosin (H&E) stain, Massion’s trichrome stain (MS) assay in cirrhotic cardiomyopathy (CCM) rat hearts. In H&E stain slides, cell nuclei are stained with blue color, other intracellular or extracellular protein are stained with pink color. The heart fibrosis can be assessed using the collagen accumulation (indicated by blue color). Normal cells are indicated by pink color in the MS assay. All heart sections were obtained from the ventricular septal of each rat.

The heart biopsy showed a large area of collagen accumulation in the CCl_4_-induced CCM rat heart. The hematoxylin and eosin (H&E) staining assay also showed cardiomyocytes presenting a disordered arrangement with more inter-space between the CCM heart cells (Figure 2). After 4 weeks of silymarin, baicalein and SHSST treatments, the cell arrangement became neat and close. Low and high dose SHSSTc treatments caused the cell arrangement to present just like the control and the collagen accumulation areas disappeared.

**Figure 3.**
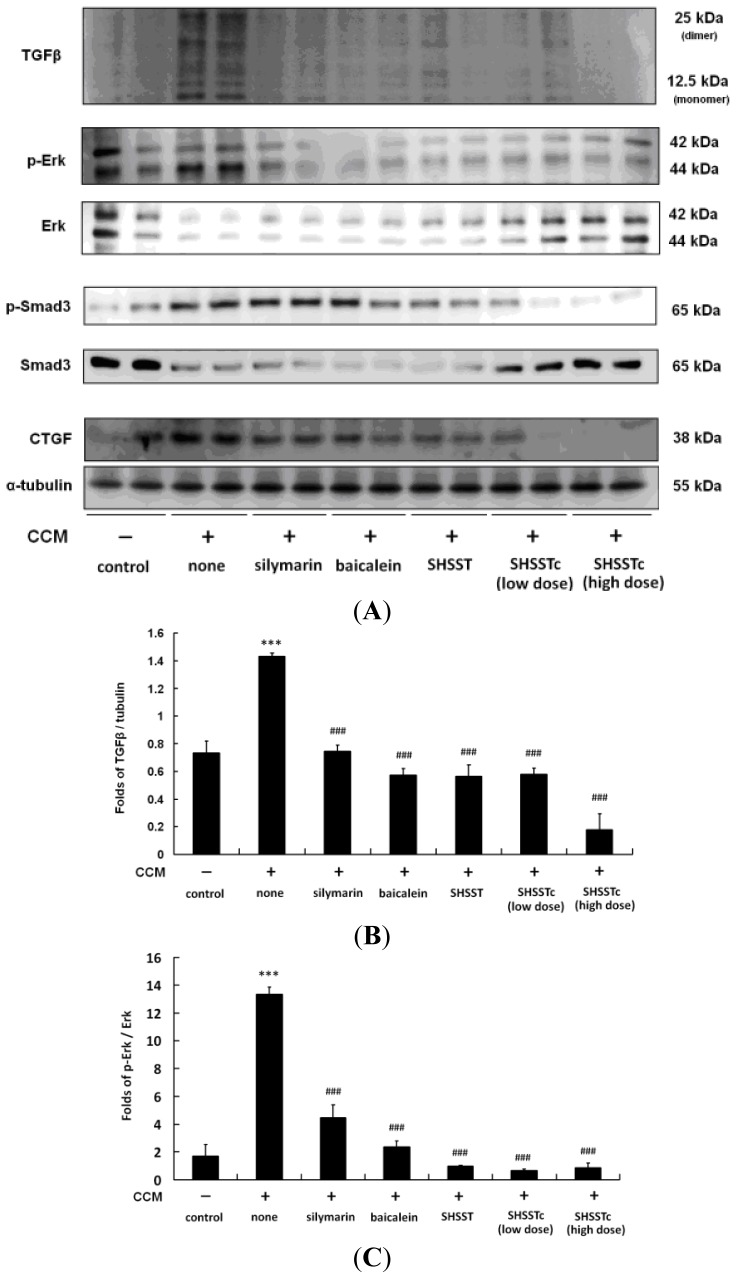

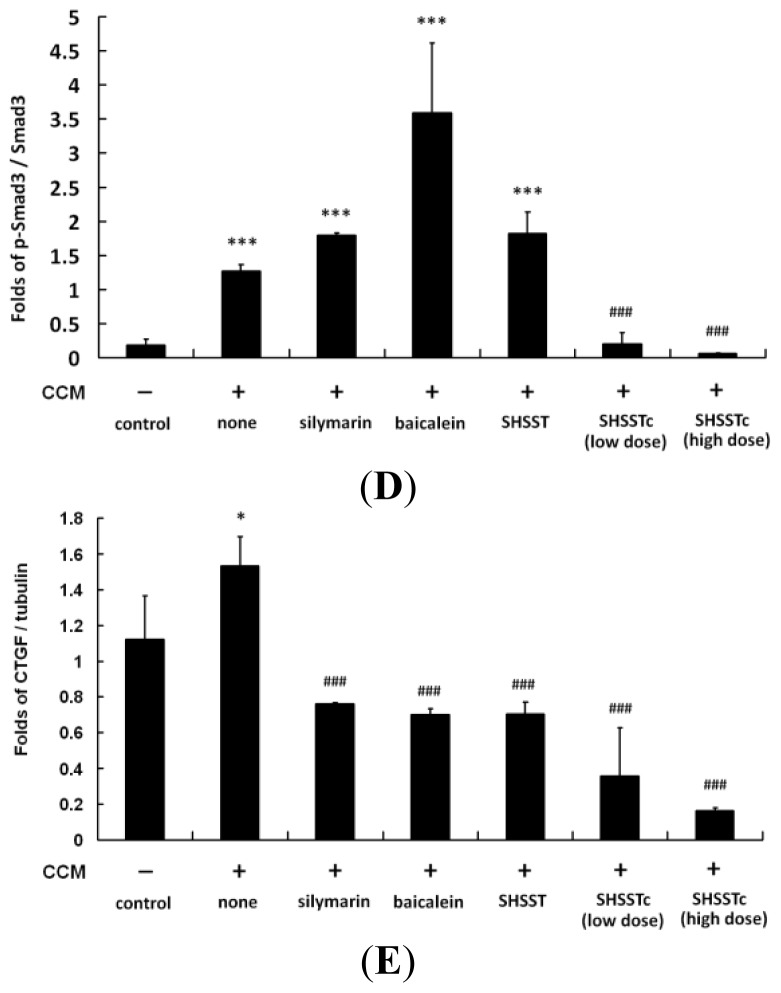
The transforming growth factor (TGF)-β pathway protein expression level analysis. (**A**) TGF-β/phoshphorylated mothers against decapentaplegic homolog 3 (Smad-3)/connective tissue growth factor (CTGF) expression levels were increased in CCM and reduced by silymarin, baicalein, SHSST, and SHSSTc low and high dose treatments; (**B**) The normalized TGF-β protein expression folds with α-tubulin; (**C**) The normalized protein expression folds of p-Erk with Erk; (**D**) The normalized protein expression folds of p-Smad3 with Smad3; (**E**) The normalized protein expression folds of CTGF with α-tubulin. *n* = 6; * *p* < 0.05, *** *p* < 0.001 compared with control group; ### *p* < 0.001 compared with the CCM group.

The protein analysis results show that CCl_4_-induced CCM efficiently increased the TGF-β, phoshphorylated mothers against decapentaplegic homolog 3 (p-Smad3) and CTGF protein levels. (Figure 3) The silymarin, baicalein and SHSST treatments can only slightly reduce the TGF-β signaling pathway. However, low and high SHSSTc dose treatments significantly reduced TGF-β pro-form (monomer) and mature-form (dimer) expression in a dose dependent manner.

Cirrhosis usually causes a hyper-dynamic state and this syndrome leads to increased cardiac output and decreased systemic vascular resistance [29]. In some CCM cases the cardiac dysfunction in cirrhosis is not associated with the liver disease severity [30]. On the contrary, one in five CCM patients suffers death after liver transplantation [31]. The death risk might be caused by the maladaptive cardiac re-circulation system with a new liver. The traceable biomarker is BNP, which is directly secreted from the cardiac response to output stress [32,33]. BNP expressions were indeed increased in the CCl_4_-induced CCM animal model in this research. (Figure 1) Cardiac hypertrophy was also induced in this animal model (Table 1).

In the results of this study cardiac fibrosis was serious in the CCM rat heart. (Figure 2) The protein analysis results also show a high mature-form of TGF-β expression in CCM. (Figure 3) Such results suggest the high expression might cause cardiac fibrosis or remodeling [11,12]. Thus, in addition to support therapy for OLT patient treatment for TGF-β expression reduction might work and needs further clinical research to confirm.

It is interesting that BNP expression levels after low and high dose SHSSTc treatments are comparable in the heart, while the fibrosis parameters (TGF-β pathway activation) are different (less activated in the high dose group). This might suggest that high dose SHSSTc treatment could provide a stronger effect on the TGF-β pathway. Another possible mechanism might exist in BNP expression reduction in CCM rat hearts after both low and high dose SHSSTc treatments.

Silymarin and baicalein (one of the bioactive compounds in SHSST) were reported to reduce TGF-β expression in CCl_4_-induced acute liver injury in a rat model [34,35]. This study also found a similar therapeutic effect can be used in CCM. SHSST treatment in CCM rats also inhibited TGF-β expression and its downstream proteins p-Smad3 and CTGF, similar to silymarin and baicalein. Although, SHSST and silymarin are effective, they are still limited by their hydrophobic characteristics. Low drug water solubility often presents poor bioavailability, preventing cirrhosis patients from accepting the normal use dose. This study used β-CD modified SHSSTc, which requires only minimal dose treatment to suppress TGF-β expression in the CCM rat heart.

SHSST was reported to have an anti-atherogenic effect (0.2 mg/mL) in human aortic smooth muscle cells and suggested to present cytokine production inhibition effects [36]. SHSST and SHSSTc were used for the first time and their cardiac protect effects against CCM evaluated in this research. The experimental results from this research show that low dose (30 mg/kg/day) SHSSTc can provide a protective effect in the CCM animal model through reducing TGF-β. SHSSTc does not just inhibit TGF-β dominated fibrosis in CCM rat hearts, but also slows CCM rat cardiac hypertrophy in a dose dependent manner. Thus, TGF-β might play an important role in heart remodeling in CCl_4_-induced CCM rat hearts. Silymarin, SHSST and baicalein treatments can reduce TGF-β expression and the downstream pathway, but SHSSTc treatment leads to a significantly improved result.

## 3. Experimental Section

### 3.1. Preparation of SHSST-β-CD (San Huang Shel Shin Tang-β-cyclodextrin) Complex and Drugs

SHSST-β-CD complex was prepared by co-precipitation. β-CD (70.0 g) was dissolved in distilled water (85 mL) at 70 °C in a water bath for 1 h. SHSST (10.0 g) in ethanol (15 mL) was slowly added to the β-CD solution with continuous agitation for 6 h. Forty milliliter of ethanol was then added drop wise to regulate the hydrophobic solute solubility in β-CD solution. Afterwards the solution was refrigerated overnight at 4 °C. The precipitated SHSSTc (SHSST:β-CD = 1:9 in weight) was recovered by filtration and washed with ethanol to remove unencapsulated SHSST. This residue was dried in a vacuum oven at −20 °C for 48 h. The final powder was stored at 4 °C until used. The silymarin and baicalein were purchased from Sigma (St. Louis, MO, USA). The silymarin, baicalein, SHSST and SHSSTc stock solutions for treatments were prepared by dissolving in distilled deionized water at 100 mg/mL each. CCl_4_ was dissolved in olive oil at the concentration of 4% *v*/*v*.

### 3.2. Animal Model

The animal use protocol was approved by the Institutional Animal Care and Use Committee (IACUC) of China Medical University (No. 100-3-B, date: 1 September 2010). There were 42 Sprague-Dawley (SD) rats purchased from BioLASCO Taiwan Co., Ltd. (Taipei, Taiwan) and divided into 7 groups (*n* = 6 each). CCl_4_ intraperitoneal (IP) injection to rats for cirrhosis induction was applied twice with 0.2 mL/kg at the first and fourteenth days. After 4 weeks from the final CCl_4_ IP injection treatments, all CCl_4_-induced CCM rat hearts were confirmed by echocardiography with the ejection fraction (EF) <50% and fractional shortening (FS) <85% parameters (control group EF > 50% and FS > 85%). After CCM induction, 4 more weeks of drug treatments were applied through gavage assay to each rat group. Groups were designated control, CCl_4_-induced CCM, silymarin (100 mg/kg/day) oral treatment, baicalein (30 mg/kg/day) oral treatment, SHSST (30 mg/kg/day) oral treatment, SHSSTc (30 mg/kg/day) oral treatment, SHSSTc (300 mg/kg/day) oral treatment.

### 3.3. Cardiac Echocardiography

M-mode echocardiographic examination was performed using a 6–15 MHz linear transducer (15–6 L) via a parasternal long axis approach. Left ventricular (LV) M-mode measurements at the papillary muscles level included left ventricular internal end-diastolic dimensions (LVIDd), left ventricular internal end-systolic dimensions (LVIDs). Fractional shortening (FS%) was calculated according to the following equation: FS% = [(LVIDd − LVIDs)/LVIDd] × 100%. Ejection fraction (EF) is defined as the ratio between the volume of blood pumped out of the LV and total volume of blood in LV.

### 3.4. Hemotoxylin and Eosin Staining

The rat hearts from each group were soaked in 10% formalin, dehydrated through graded alcohols and embedded in paraffin wax. The 0.2 μm-thick paraffin sections were then cut into slices from these paraffin-embedded tissue blocks. The tissue sections were deparaffinized by immersing in xylene and rehydrated. All slices were dyed with hematoxylin and eosin (H&E) and then rinsed with water. Each slide was dehydrated through graded alcohols. Heart sections were finally soaked in xylene twice. Photomicrographs were obtained using Zeiss Axiophot microscopes (Taiwan Instrument Co., Taipei, Taiwan).

### 3.5. Masson’s Trichrome Staining

The rat hearts from each group were soaked in 10% formalin, dehydrated through graded alcohols and embedded in paraffin wax. The 0.2 μm-thick paraffin sections were then cut into slices from these paraffin-embedded tissue blocks. The tissue sections were deparaffinized by immersing in xylene and rehydrated. Samples were then stained with Masson’s trichrome staining to investigate liver histo-logical and fibrotic changes. Photomicrographs were obtained using Zeiss Axiophot microscopes.

### 3.6. Tissue Protein Extraction

Heart tissue extracts from 6 rats in each group were obtained by homogenizing in a lysis buffer (0.05 M Tris–HCl, pH 7.4, 0.15 M NaCl, 0.25% deoxycholic acid, 1% nonyl phenoxypolyethoxylethanol, 1 mM EDTA) at a ratio of 100 mg tissue/1 mL buffer. The homogenates were placed on ice and then centrifuged at 13,000 rpm for 40 min. The supernatants were collected and stored at −80 °C for further experiments.

### 3.7. Western Blot Assay

Heart tissue protein concentration extracts were determined using the Lowry protein assay. Protein samples were separated in a 12% SDS polyacrylamide gel electrophoresis (SDS-PAGE) with a constant voltage of 75 V for 120 min. Proteins were then transferred to Hybond-C membranes (GE healthcare UK Ltd., Little Chalfont, Buckinghamshire, UK) using 50 V for 3 h. Polyvinylidene difluoride (PVDF) membranes were incubated in 3% bovine serum albumin (BSA) in tricine buffer solution. Primary antibodies including brain natriuretic peptide (BNP, SC-18818, Santa Cruz Biotechnology, Dallas, TX, USA), TGF-β (SC-31609, Santa Cruz Biotechnology), Smad-3 (SC-8332, Santa Cruz Biotechnology), α-tubulin (SC-5286, Santa Cruz Biotechnology), CTGF (SC-14939, Santa Cruz Biotechnology), were added into the membranes for recognizing the fitted proteins. Horseradish peroxidase-labeled antibodies were finally used and pictures were then taken with Fujifilm LAS-4000 (GE healthcare UK Ltd.).

### 3.8. Statistical Analysis

The results shown are the means ± SD of three independent experiments. Statistical analysis was performed using one-way analysis of variants. The Student’s *t* test was applied for paired samples.

## 4. Conclusions

The experimental evidence reported here suggests that SHSSTc treatment can improve the present clinical treatment for non-cirrhotic heart failure. Present clinical treatment includes bed rest, oxygen and diuretics. TGF-β expression elimination in CCM through SHSSTc treatment can improve heart function in CCM and may also improve the survival rate for OLT patients after clinical liver transplantation surgery.

## Figures and Tables

**Table 1.** Cardio vascular structure physiological characteristics assessment.

Groups	Control	CCM	CCM silymarin	CCM Baicalein	CCM SHSST	CCM SHSSTc (low dose)	CCM SHSSTc (high dose)
BW (g)	323.0 ± 8.9	352.3 ± 14.0 *	343.7 ± 6.1 *	325.7 ± 11.6	366.0 ± 22.3 *	349.7 ± 25.8	316.7 ± 9.1 #
TL (cm)	3.80 ± 0.15	4.10 ± 0.00	3.97 ± 0.06	4.10 ± 0.00	4.10 ± 0.10	4.03 ± 0.06	4.00 ± 0.10
HW (mg)	936.7 ± 53.3	1146.0 ± 21.9 **	927.3 ± 49.8 ###	921.7 ± 17.8 ###	940.3 ± 25.1 ###	944.0 ± 20.1 ###	965.7 ± 11.2 ###
LVW (mg)	681.7 ± 15.0	922.7 ± 11.9 ***	711.3 ± 62.5 ##	720.0 ± 62.2 ##	683.7 ± 52.5 ##	687.3 ± 54.7 ##	683.0 ± 22.7 ###
HW/BW (mg/g)	2.90 ± 0.10	3.26 ± 0.15 *	2.70 ± 0.19 ##	2.83 ± 0.13 #	2.85 ± 0.22 #	2.71 ± 0.25 #	3.15 ± 0.12
HW/TL (mg/cm)	238.6 ± 20.5	279.5 ± 5.3 *	233.9 ± 16.0 ##	224.8 ± 4.3 ###	22.9 ± 11.7 ##	234.1 ± 8.2 ##	241.6 ± 8.3 ##
LVW/BW (mg/g)	2.1 ± 0.1	2.6 ± 0.1 **	2.1 ± 0.2 ##	2.2 ± 0.3	1.9 ± 0.3 ##	2.0 ± 0.3 #	2.2 ± 0.0 ##
LVW/HW (mg/mg)	0.73 ± 0.03	0.81 ± 0.01 *	0.77 ± 0.03	0.78 ± 0.05	0.73 ± 0.04 #	0.73 ± 0.04 #	0.71 ± 0.03 ##
LVW/TL (mg/cm)	173.5 ± 8.1	225.0 ± 2.9 ***	179.5 ± 15.1 #	175.6 ± 15.2 ##	167.0 ± 16.9 ##	170.6 ± 15.8 ##	170.7 ± 2.4 ###
EF (%)	80 ± 2.6	68.5 ± 1.0 ***	72.8 ± 3.3 ##	72.3 ± 1.5 ###	74 ± 1.0 ###	76.7 ± 1.5 ###	77 ± 0.3 ###
FS (%)	43.7 ± 2.1	33.7 ± 1.4 ***	38.8 ± 6.1	36.5 ± 1.0 ##	38.3 ± 0.6 ###	40.7 ± 1.5 ###	41.0 ± 0.7 ###
LVIDd (mm)	8.5 ± 0.1	8.0 ± 0.2 ***	8.2 ± 0.4	8.1 ± 0.3	8.1 ± 0.3	8.3 ± 0.2 ##	8.4 ± 0.1 ###

BW, body weight; TL, tibia length; HW, whole heart weight; LVW, left vestibular weight; EF, ejection fraction; FS, fractional shortening; LVIDd, left ventricular interior dimention;

* *p* < 0.05,

** *p* < 0.01,

*** *p* < 0.001 compared with control group;

# *p* < 0.05,

## *p* < 0.01,

### *p* < 0.001 compared with cirrhotic cardiomyopathy (CCM) group.
